# Differential gene expression in cisplatin-resistant and -sensitive testicular germ cell tumor cell lines

**DOI:** 10.18632/oncotarget.27844

**Published:** 2020-12-22

**Authors:** Jan Roška, Lenka Wachsmannová, Lenka Hurbanová, Zuzana Šestáková, Thomas Mueller, Dana Jurkovičová, Miroslav Chovanec

**Affiliations:** ^1^Department of Genetics, Cancer Research Institute, Biomedical Research Center, Slovak Academy of Sciences, Bratislava, Slovak Republic; ^2^University Clinic for Internal Medicine IV, Hematology/Oncology, Medical Faculty of Martin Luther University Halle-Wittenberg, Halle (Saale), Germany; ^*^Co-senior authors

**Keywords:** gene expression array, cisplatin, testicular germ cell tumors, prognostic biomarkers, pluripotency factors

## Abstract

Testicular germ cell tumors (TGCTs) represent a well curable malignity due to their exceptional response to cisplatin (CDDP). Despite remarkable treatment results, approximately 5% of TGCT patients develop CDDP resistance and die. Exceptional curability makes TGCTs a highly valuable model system for studying the molecular mechanisms of CDDP sensitivity. Our study was aimed at revealing difference in gene expression between the CDDP-resistant and -sensitive TGCT cell lines, and hence at identifying candidate genes that could serve as potential biomarkers of CDDP response. Using gene expression array, we identified 281 genes that are differentially expressed in CDDP-resistant compared to -sensitive TGCT cell lines. The expression of 25 genes with the highest fold change was validated by RT-qPCR. Of them, *DNMT3L*, *GAL*, *IGFBP2*, *IGFBP7*, *L1TD1*, *NANOG*, *NTF3*, *POU5F1*, *SOX2*, *WNT6*, *ZFP42*, *ID2*, *PCP4*, *SLC40A1* and *TRIB3*, displayed comparable expression change in gene expression array and RT-qPCR, when all CDDP-resistant TGCT cell lines were pairwise combined with all -sensitive ones. Products of the identified genes are pluripotency factors, or are involved in processes, such as cell metabolism, proliferation or migration. We propose that, after clinical validation, these genes could serve as prognostic biomarkers for early detection of CDDP response in TGCT patients.

## INTRODUCTION

Testicular germ cell tumors (TGCTs) are the most common solid malignity in men with an ever-increasing incidence. Its highest incidence occurs in young men, with an average age of diagnosis of 36 years [1–3]. TGCTs are characterized as the most sensitive malignity to cisplatin (CDDP)-based chemotherapy, making them a very valuable model system for studying the molecular mechanisms underlying CDDP response [4]. Up to 80% of patients, even in advanced metastatic stage, can be cured with standard first-line CDDP-based chemotherapy. However, about 20% of patients relapse as a consequence of an inadequate/aberrant response to chemotherapy. The relapsed patients may be cured by salvage chemotherapy. Despite excellent treatment outcome, approximately 5% of patients do not respond even to salvage chemotherapy and die due to an adverse treatment response or develop chemoresistance [5–7].

In terms of classification, TGCTs are a heterogeneous group of malignity mainly due to pluripotent properties of the testicular germ cells, whom they originate from [8]. They are derived from abnormally differentiated testicular germ cells which have acquired susceptible genetic changes. During childhood and adolescence, they occur in the precursor stage referred to as germ cell neoplasia *in situ* of the testis, after which they change to invasive TGCTs [9]. Histologically, TGCTs are classified into two main subgroups, seminomas (SEs) and non-seminomas (NSEs) [10]. SEs account for 55% of TGCTs, are homogenous, and less aggressive. In contrast, NSEs are heterogeneous, contain multiple histological components and show higher aggressiveness. They exhibit embryonic and extra-embryonic differentiation patterns with different phenotypes, embryonal carcinoma (EC), choriocarcinoma (CC), yolk sac tumor (YST) and teratoma (TE) (reviewed in [8, 11–13]).

There are multiple mechanisms contributing to cellular response to CDDP, such as transport systems, interactions of the drug with various intracellular components, DNA damage response (DDR) and repair, cell cycle, senescence, and apoptosis [14]. Since the primary pharmacological target of CDDP is DNA molecule, cytotoxicity of this agent is mainly manifested through induction of DNA damage [15, 16]. Therefore, correlation between the CDDP sensitivity and expression level of the DDR and repair factors, particularly those involved in nucleotide excision repair (NER), has attracted research attention. Indeed, up-regulation of the NER proteins in various tumor types has repeatedly been associated with a worse prognosis of patients, with decreased NER levels leading to an improved response to CDDP-based chemotherapy and patients’ survival [17–21]. In TGCTs, however, data are far from being consistent [22–27], and are still awaiting in-depth consolidation.

Currently, most commonly used TGCT markers are serum levels of α-fetoprotein (AFP), β-subunit of the human chorionic gonadotropin, and lactate dehydrogenase. They are usually used at the stage of diagnosis [28]. In addition, a prognostic factor-based staging system for metastatic TGCTs, established by the International Germ Cell Cancer Cooperative Group (IGCCCG), considers pre-chemotherapy levels of these serum markers as independent prognostic variable alongside with histological type of primary tumor, primary tumor location, and the presence of non-pulmonary visceral metastases, to categorize patients to good, intermediate, or poor prognosis group [29]. However, IGCCCG criteria are not adequate to predict patient outcome precisely, and significant variability exists in the prognosis of patients within the same IGCCCG risk group. Therefore, reliable predictive markers are urgently needed to identify poor prognosis TGCT patients (refractory or those with high risk for relapse) most likely to be disadvantaged from standard first-line CDDP-based chemotherapy and, instead, allowing their early management through more aggressive or experimental therapy.

As multiple factors are involved in CDDP response, high-throughput methods represent a very powerful tool contributing to their discovery. In addition, availability of experimental *in vitro* models displaying a broad spectrum of CDDP response further extends toolbox required for detailed understanding of processes underlying CDDP toxicity. In case of TGCTs, such cell lines (covering all known histological sub-types) had been established over the years and, in combination with genome- and proteome-wide methods, already contributed to revealing of numerous CDDP response factors [30–32]. Nevertheless, huge gaps in our understanding of CDDP response still exist, and therefore the present study was aimed to potentially increase the total number of the CDDP response factors through the gene expression profiling in the unique sets of CDDP-resistant and -sensitive TGCT cell lines.

## RESULTS

### Gene expression profiles in TGCT cell lines

**Figure 1:**
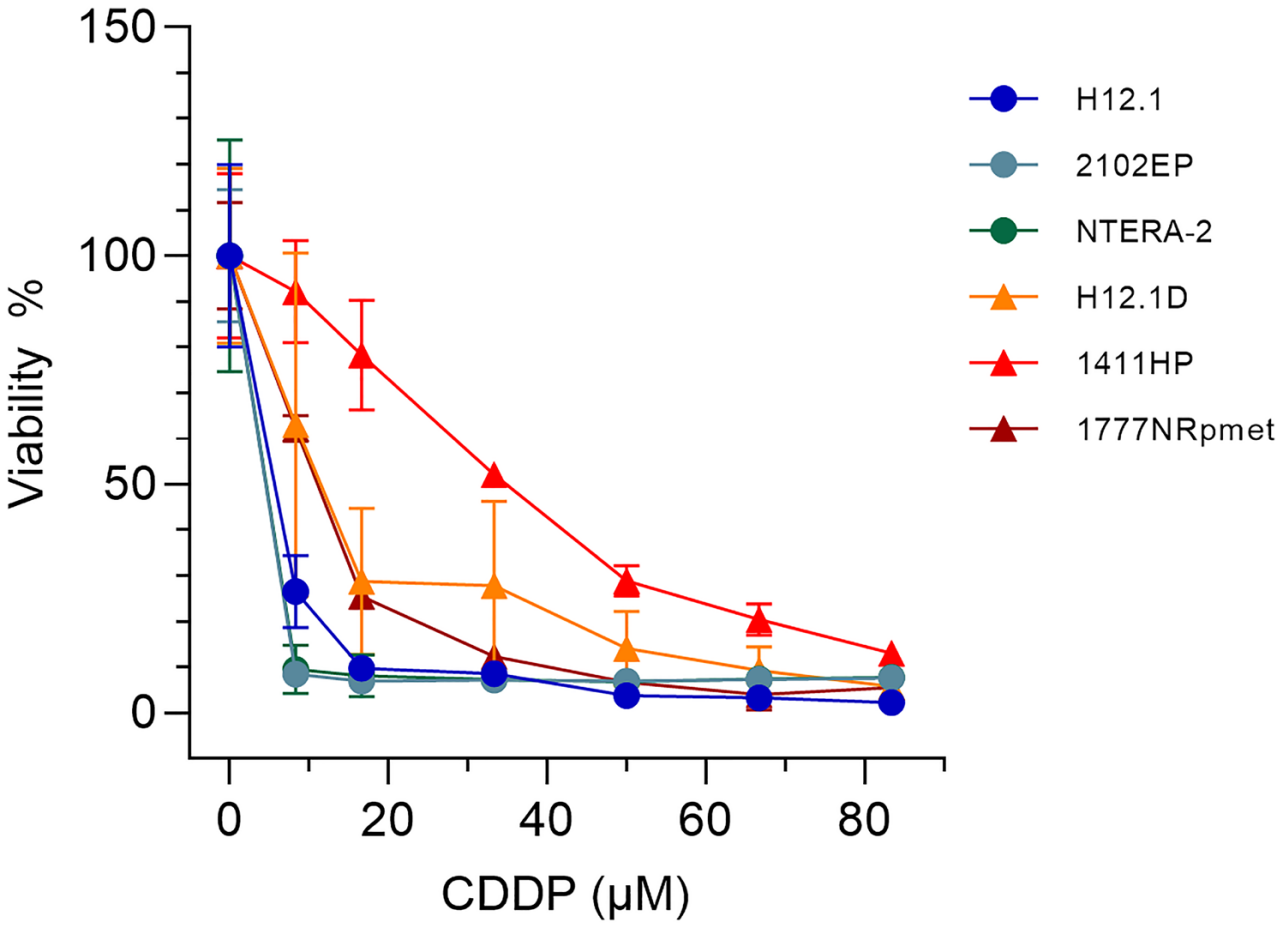
Survival of TGCT cell lines used in this study after CDDP treatment. H12.1, 2102EP, NTERA-2, H12.1D, 1411HP and 1777NRpmet TGCT cell lines were exposed to the increasing concentrations of CDDP for 24 hr in culture medium and survival was estimated by the MTT test. The obtained curves were used to calculate the IC_50_ values shown in Table 1.

TGCT cell lines of different origin and histological type displaying a diverse level of CDDP sensitivity were used (Table 1). Cell lines selection was based on the mean 50% and 90% inhibitory CDDP concentrations (IC_50_ and IC_90_ values, respectively) demonstrated in our previous studies [33, 34], as well as on the IC_50_ value examined in the present study (Table 1 and Figure 1). Although there seems to be discrepancy between the IC_50_ values when study by Schaffrath and co-workers [33] is compared to our, a plausible explanation lies in different CDDP treatment conditions and methods used to determine CDDP cytotoxicity: while the previous study used 96 hr CDDP treatment and the sulforhodamin-B-based assay, we treated TGCT cell lines with CDDP for 24 hr and determined cytotoxicity using the tetrazolium salt (3-(4,5-dimethylthiazol-2-yl)-2,5-diphenyltetrazolium bromide; MTT)-based assay (see section Materials and Methods). Importantly, and in accordance to previous findings, H12.1D [34], 1411HP [35] and 1777NRpmet [36] (collectively referred to as CDDP-resistant) TGCT cell lines are clearly more resistant to CDDP than H12.1 [37], 2102EP [38–41] and NTERA-2 [42–44] (collectively referred to as CDDP-sensitive) cell lines (Table 1).

**Table 1:** Characterization of TGCT cell lines used in this study

Cell line	Histological type	CDDP response	IC_50_ (μM) [33]	IC_90_ (μM) [34]	IC_50_ (μM)^a^	Reference
H12.1	Established from primary TGCT, displaying morphology of EC	Sensitive	0.5 ± 0.11	3	6.87 ± 2.43	[37]
2102EP	Derived from primary tumour classified as TE with YST, with cells also resembling EC	Sensitive	ND	3	5.78 ± 1.14	[38–41]
NTERA-2	Malignant pluripotent EC, clonally derived from Tera-2	Sensitive	ND	ND	5.63 ± 1.92	[42–44]
H12.1D	EC, *in vitro* differentiation medium-induced derivate of H12.1	Resistant	10.45 ± 3.28	ND	13.34 ± 8.21	[34]
1411HP	Established from a patient with metastatic testicular cancer, showing combined EC and YST	Resistant	4.70 ± 0.44	10	34.93 ± 1.04	[35]
1777NRpmet	Derived from a retroperitoneal lymph node metastasis showing differentiated EC with immature TE	Resistant	1.64 ± 0.74	16	11.11 ± 0.23	[36]

ND: not determined in quoted reference, TGCT: testicular germ cell tumour, EC: embryonal carcinoma, TE: teratoma, YST: yolk sac tumour. ^a^ this study.

With the exception of NTERA-2, detailed information on TGCT cell lines (such as original tumor, xenograft tumor *etc*.) used in this study was provided in our previous study [34]. Cell lines, H12.1, 2102EP and 1411HP, can grow as subcutaneous xenograft tumors in nude mice in accordance to the original descriptions. Furthermore, H12.1 tumors respond to CDDP therapy with regressions, whereas 1411HP progresses and displays only some degree of growth retardation [45]. 2102EP tumors are even more sensitive than H12.1 tumors and show partly complete regression since these cells can not differentiate and the tumor only contains EC cells, whereas H12.1 cells can differentiate and the tumors are typically composed of EC with YST, CC and TE differentiation representing a typical NSE (our unpublished data). The 1777NRpmet cell line was derived from a retroperitoneal metastasis and classified histologically as EC [36], but does not grow well as xenograft tumor in our experiments (unpublished data). This is likely due to differentiation in more somatic lineages similar to H12.1D, which, however, was *in vitro* differentiated from H12.1 and does not grow as xenograft tumor. Both cell lines show a lack of expression of the embryonal transcription factor OCT4 [34]. In contrast, 1411HP tumors, also lacking *OCT4* expression, show differentiation towards extra-embryonal YST [46]. The differentiation aspect is further described in the discussion section.

To disclose the genes that are differentially expressed in CDDP-resistant TGCT cell lines compared with -sensitive ones, various pairwise combinations of cell lines were examined. Since H12.1 and H12.1D TGCT cell lines represent an authentic isogenic pair, a priority was given to data acquired for this particular pairwise combination, even though the H12.D cell line was not directly made to be resistant to CDDP. Nevertheless, a mechanism of CDDP resistance in this cell line, achieved by cultivation in differentiation-inducing medium leading to the loss of *POU5F1* expression [34], parallels at the molecular level the one induced directly by CDDP treatment, as demonstrated by the fact that CDDP induces resistance to itself by triggering a differentiation response *via* decrease in the expression of *POU5F1* in pluripotent germ cell tumor (GCT) cells [47]. Other pairs combine non-isogenic cell lines, but data obtained for them can also be valuable, potentially addressing other aspects of TGCT biology related to CDDP response. In line with this statement, a huge overlap in differentially expressed genes between isogenic and non-isogenic pairwise combinations was obtained. In addition, metastatic origin and chemotherapy resistant phenotype of 1411HP and 1777NRpmet cell lines (Table 1) allowed revealing the genes related to acquired mechanism of CDDP resistance and metastatic phenotype. Finally, analysis comparing all CDDP-resistant TGCT cell lines with all -sensitive ones reflects a histological heterogeneity of the disease that is represented by a prevalent number of mixed tumors.

**Figure 2:**
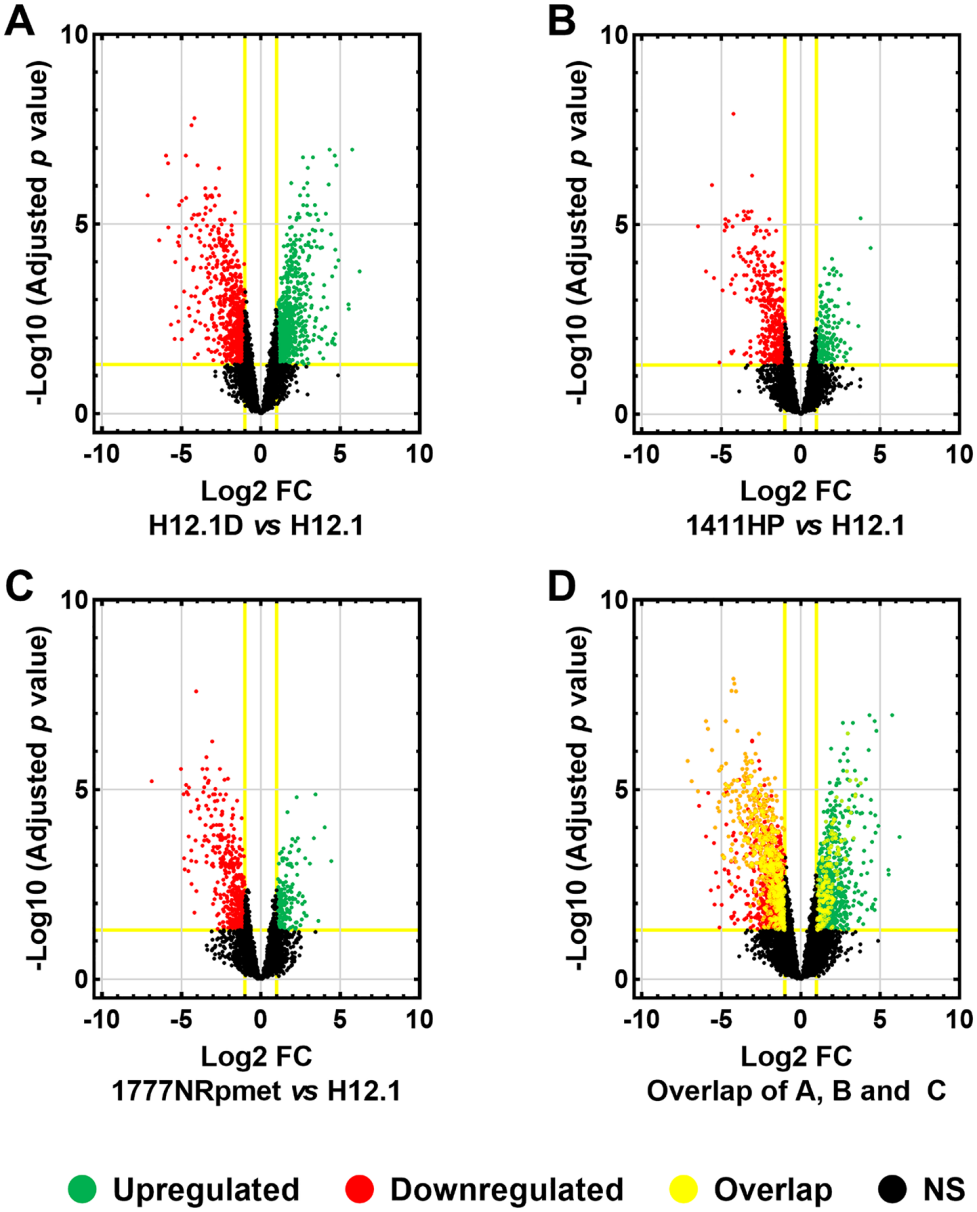
Differentially expressed genes in TGCT cell lines. Differentially expressed genes in CDDP-resistant TGCT cell lines were compared with -sensitive H12.1 cell line. Volcano plot comparisons of mean log2-FC of gene expression between H12.1D and H12.1 (**A**), 1411HP and H12.1 (**B**) and 1777NRpmet and H12.1 (**C**) cell lines. Overlap of all three CDDP-resistant TGCT cell lines vs H12.1 (**D**). Vertical lines correspond to > 2.0 up-regulation and < 0.5 down-regulation. Horizontal line represents a threshold of adjusted *p* < 0.05. Green and red points in the volcano plot represent differentially expressed genes that are statistically significant and > 2.0 up- and < 0.5 down-regulated. Black points represent gene expression which was not statistically significant, nor showed > 2.0 up-regulation or < 0.5 down-regulation. Yellow rims around individual points show genes which had an overlap in all CDDP-resistant TGCT cell lines with 2-FC in expression and an adjusted *p* < 0.05.

First, we profiled gene expression in CDDP-resistant (H12.1D, 1411HP and 1777NRpmet) and -sensitive (H12.1) TGCT cell lines to identify differentially expressed genes between the H12.1D vs H12.1, 1411HP vs H12.1, and 1777NRpmet vs H12.1 pairwise cell line combinations. After visualization of microarray signal, differential gene expression was compared. Differences in gene expression (represented as the mean fold change; FC) in CDDP-resistant vs -sensitive TGCT cell lines were considered to be significant, when adjusted *p* value was < 0.05. When compared H12.1D with H12.1, statistically significant difference in expression of 2,226 genes was found. Of them, 1,158 genes were down-regulated and 1,068 up-regulated (Figure 2A and Supplementary Table 1). When 1411HP cell line was compared with H12.1, expression of 908 genes was significantly changed, with 636 genes being down-regulated and 272 genes being up-regulated (Figure 2B and Supplementary Table 2). Finally, 1777NRpmet vs H12.1 comparison brought statistical difference in expression of 839 genes, of which 252 and 587 were up- and down-regulated, respectively (Figure 2C and Supplementary Table 3).

### Validation of expression of candidate genes

**Figure 3:**
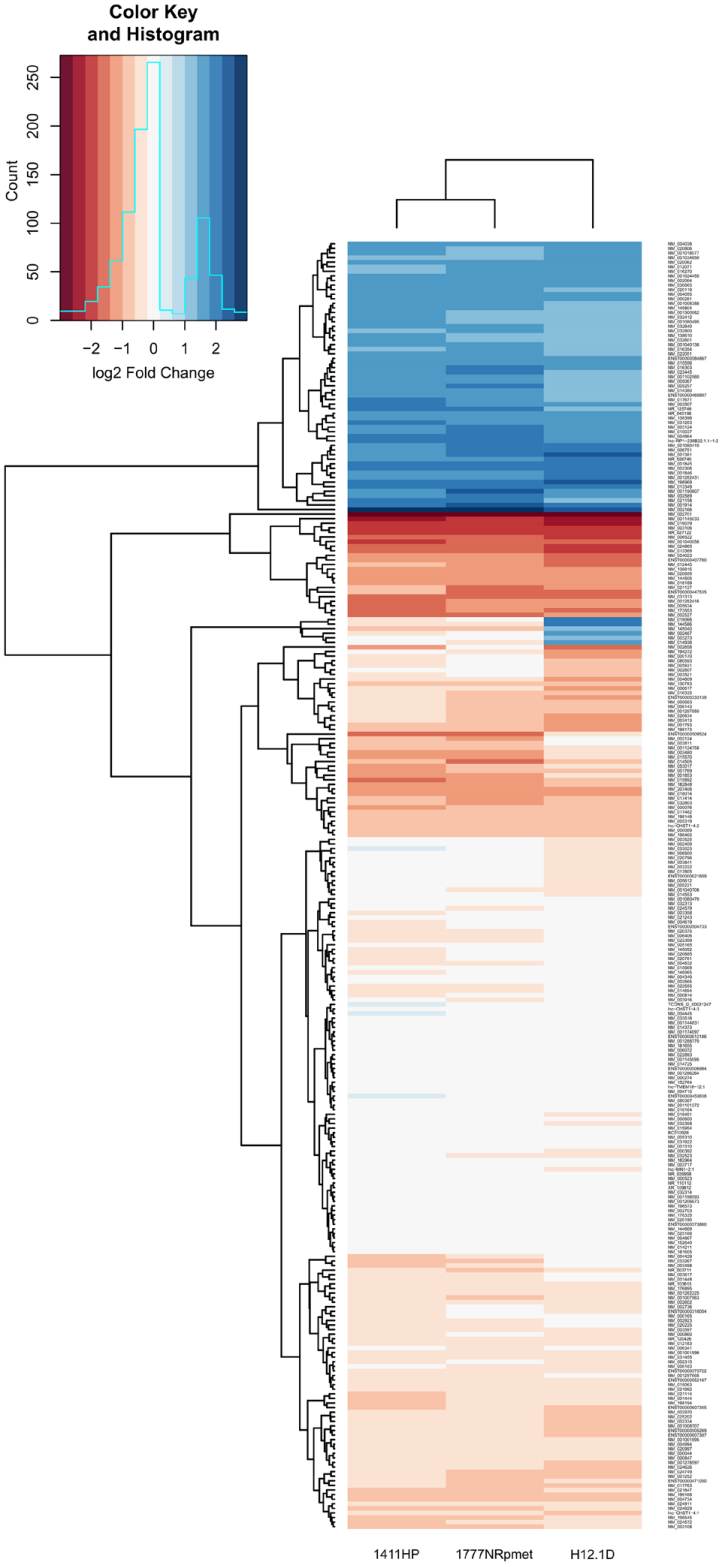
Differentially expressed genes in CDDP-resistant TGCT cell lines. Heatmap illustrates differentially expressed genes with log2 transformed mean relative expression > 2 and *p* < 0.05 in CDDP-resistant TGCT cell lines in comparison with -sensitive H12.1 TGCT cell line using hierarchical clustering. Red color indicates low relative expression and blue color indicates high relative expression.

Parameters for mean log FC > 2.0 and < 0.5 for up- and down-regulated expression change were further introduced respectively, to narrow the gene data set with *p* < 0.05. This set-up disclosed 760 up- and 773 down-regulated genes for H12.1D (Supplementary Table 4), 187 up- and 486 down-regulated genes for 1411HP (Supplementary Table 5), and 183 up- and 451 down-regulated genes for 1777NRpmet (Supplementary Table 6), when compared with H12.1. After overlapping the obtained data sets, 281 genes displayed significant differential expression in all CDDP-resistant TGCT cell lines when compared with H12.1 -sensitive cell line (Figure 2D and Supplementary Table 7). Of them, 275 genes were comparably down- or up-regulated, while the remaining 6 genes (*MAGEL2*, *LYPD1*, *NDN*, *ENPP4*, *PRKCDBP* and *TM7SF2*) displayed up-regulation in H12.1D, but down-regulation in both 1411HP and 1777NRpmet (Table 2). Figure 3 shows expression profiles of down- and up-regulated genes with log2-FC with the mean FC > 2.0 vs CDDP-sensitive H12.1 TGCT cell line.

**Table 2:** List of genes that displayed statistically significant up-regulation in primary tumour-derived (H12.1D), but down-regulation in metastasis-derived (1411HP and 1777NRpmet), CDDP-resistant TGCT cell lines compared to -sensitive H12.1

Gene (aliases)	Molecular/biological function of the protein
*MAGEL2 (PWLS, nM15, NDNL1, SHFYNG)*	*MAGE family member L2* Prader-Willi syndrome (PWS) is manifested by neonatal hypotonia, developmental delay and childhood-onset obesity. Necdin (NDN), a gene involved in the terminal differentiation of neurons, has been implicated as one of the genes responsible for the etiology of PWS. The *MAGEL2* gene is structurally similar to NDN, is localized to the PWS chromosomal region and paternally imprinted, and therefore it has a possible role in PWS.
*LYPD1 (PHTS, LYPDC1)*	*LY6/PLAUR domain containing 1* It may function in a new and p53-independent tumour suppressor pathway.
*NDN (PWCR, HsT16328)*	*Necdin, MAGE family member* The gene is located in the PWS deletion region. It is an imprinted gene and is expressed exclusively from the paternal allele. The protein encoded by this gene suppresses growth in postmitotic neurons.
*ENPP4 (NPP4)*	*Ectonucleotide pyrophosphatase/phosphodiesterase 4* It is a type I extracellular membrane protein on brain vascular endothelium inducing platelet aggregation *via* the hydrolysis of Ap3A.
*PRKCDBP (SRBC, HSRBC, CAVIN3, cavin-3)*	*Protein kinase C delta-binding protein* The expression of this protein in cultured cell lines is strongly induced by serum starvation. This protein was found to be down-regulated in various cancer cell lines, suggesting its possible tumour suppressor function.
*TM7SF2 (ANG1, C14SR, DHCR14A, NET47)*	*Transmembrane 7 superfamily member 2* The protein is 3β-hydroxysterol δ(14)-reductase, responsible for the reduction of C14-unsaturated sterols in cholesterol biosynthesis. The *TM7SF2* gene expression is controlled by cell sterol levels.

Source: https://www.ncbi.nlm.nih.gov/genome/guide/human/.

Datasets shown in Supplementary Tables 4, 5 and 6 were used to select candidate genes for validation by RT-qPCR. In total, 25 genes were selected. Criteria for selection of these genes were based on their appearance in at least two out of three mentioned datasets and their as highest expression change as possible. Notably, and expectedly, expression changes of the selected genes detected by RT-qPCR paralleled the ones obtained by gene expression array, although when CDDP-resistant TGCT cell lines 1411HP and 1777NRpmet were pairwise compared with two other CDDP-sensitive cell lines, 2102EP and NTERA-2, it slightly differed, depending on CDDP-sensitive cell line they were normalized against. Therefore, we compared expression of the validated genes only for a pairwise combination H12.1D vs H12.1, as H12.1D cell line is a true isogenic derivative of H12.1. To show high degree of similarity and to simplify data interpretation, we divided the validated genes into two groups, 13 genes that were significantly down-regulated in original gene expression array data set (*C11orf96*, *DNMT3L, GAL*, *IGDCC3*, *IGFBP2*, *IGFBP7*, *L1TD1*, *NANOG*, *NTF3*, *POU5F1*, *SOX2*, *WNT6* and *ZFP42*) and 12 up-regulated genes (*BEX2*, *CCN1*, *CYB5A*, *CYB5R2*, *FADS2*, *ID2*, *ISG20*, *PCP4*, *REC8*, *SLC40A1, TMSB4X* and *TRIB3*). Supplementary Table 8 lists these genes along with brief molecular and biological characterization of their products.

### Up-regulated genes

**Figure 4:**
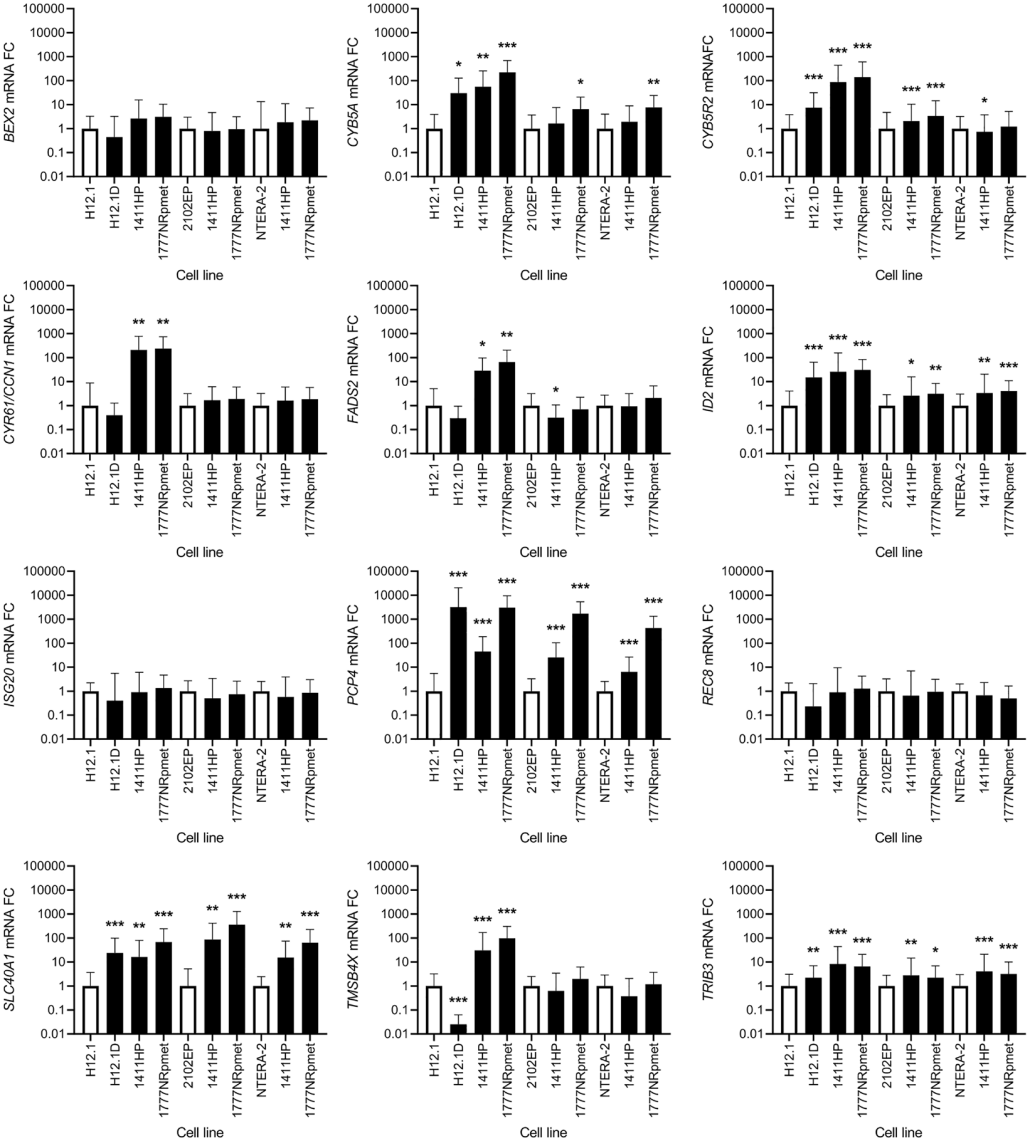
Expression change of 12 candidate genes that were originally identified as up-regulated using gene expression array. CDDP-resistant (H12.1D, 1411HP and 1777NRpmet) and -sensitive (H12.1, 2102EP and NTERA-2) TGCT cell lines are represented as black and white bars, respectively. Error bars represent upper and lower limits of expression of three technical and three biological replicates. ^*^ *p* ≤ 0.05, ^**^ *p* ≤ 0.01, ^***^ *p* ≤ 0.001.

When comparing CDDP-resistant TGCTs cell lines with H12.1 -sensitive control, we observed significant up-regulation of the *TRIB3*, *ID2*, *PCP4*, *CYB5A*, *CYB5R2* and *SLC40A1* genes in all CDDP-resistant cell lines. *CCN1* was significantly up-regulated in 1411HP and 1777NRpmet cell lines (*p* < 0.001), but not in H12.1D (*p* = 0.088). *ISG20*, *REC8* and *BEX2* did not display statistically significant up-regulation of their expression in any CDDP-resistant cell line compared with H12.1 (*p* = 0.823, 0.401 and 0.059 for 1411HP, 1777NRpmet and H12.1D, respectively). *FADS2* was significantly up-regulated in both 1411HP and 1777NRpmet cell lines and showed a trend of down-regulation in H12.1D, as compared with H12.1. This gene was also significantly down-regulated in 1411HP when compared with 2102EP CDDP-sensitive cell line. *TMSB4X* was down-regulated in CDDP-resistant H12.1D cell line when pairwise combined with H12.1, even though in 1411HP and 1777NRpmet it was up-regulated. Significantly up-regulated expression of *ID2*, *PCP4*, *TRIB3*, *SLC40A1* and *CYB5R2* was revealed when 1411HP and 1777NRpmet CDDP-resistant cell lines were compared with 2102EP. *CYB5A* was significantly up-regulated in 1777NRpmet when pairwise combined with 2102EP (*p* = 0.014). Neither 1411HP nor 1777NRpmet displayed any significant change in expression of the *TMSB4X*, *BEX2*, *CCN1*, *REC8* and *ISG20* genes when compared with 2102EP. When 1411HP and 1777NRpmet cell lines were pairwise combined with NTERA-2, significant change of the *TRIB3*, *PCP4*, *ID2* and *SLC40A1* gene expression was observed. When analyzed against NTERA-2, *CYB5R2* was significantly down-regulated in 1411HP (*p* = 0.047) and *CYB5A* was up-regulated in 1777NRpmet (*p* = 0.008). Neither 1411HP nor 1777NRpmet demonstrated any significant change in expression of the *FADS2*, *REC8*, *CCN1*, *BEX2* and *TMSB4X* genes when compared with NTERA-2 (Figure 4). Individual statistical significance for all 25 validated genes is illustrated in Table 3.

**Table 3:** Statistical analysis of expression for the 25 most differentially expressed genes

Validated gene	*p* value						
	H12.1			2102EP		NTERA-2	
	H12.1D	1411HP	1777NRpmet	1411HP	1777NRpmet	1411HP	1777NRpmet
*BEX2*	NS	NS	NS	NS	NS	NS	NS
*CYB5A*	0.0185	0.0036	< 0.001	NS	0.0138	NS	0.0079
*CYB5R2*	< 0.001	< 0.001	< 0.001	< 0.001	< 0.001	0.047	NS
***PCP4***	< 0.001	< 0.001	< 0.001	< 0.001	< 0.001	< 0.001	< 0.001
*ISG20*	NS	NS	NS	NS	NS	NS	NS
*CCN1*	NS	0.0043	0.0013	NS	NS	NS	NS
*REC8*	NS	NS	NS	NS	NS	NS	NS
***TRIB3***	0.0051	< 0.001	< 0.001	0.0016	0.0138	< 0.001	< 0.001
*FADS2*	0.015	< 0.001	< 0.001	0.035	NS	NS	NS
***ID2***	< 0.001	< 0.001	< 0.001	0.0310	0.0033	0.0050	< 0.001
*TMSB4X*	< 0.001	< 0.001	<0.001	NS	NS	NS	NS
***SLC40A1***	< 0.001	0.0021	< 0.001	0.0095	< 0.001	0.0042	< 0.001
***IGFBP2***	< 0.001	< 0.001	< 0.001	0.0069	< 0.001	0.0069	< 0.001
*IGFBP7*	0.0012	< 0.001	< 0.001	<0.001	NS	< 0.001	0.0162
***L1TD1***	< 0.001	0.0018	< 0.001	0.0042	< 0.001	0.0266	< 0.001
*NTF3*	< 0.001	< 0.001	< 0.001	NS	NS	NS	0.0036
***NANOG***	< 0.001	< 0.001	0.001	< 0.001	< 0.001	< 0.001	0.0029
***POU5F1***	< 0.001	< 0.001	< 0.001	< 0.001	< 0.001	< 0.001	< 0.001
***SOX2***	< 0.001	0.0117	< 0.001	0.0033	< 0.001	0.0073	< 0.001
*C11orf96*	0.0199	NS	NS	NS	NS	NS	NS
*DNMT3L*	< 0.001	< 0.001	< 0.001	< 0.001	< 0.001	NS	NS
*GAL*	0.0039	< 0.001	NS	< 0.001	< 0.001	< 0.001	NS
*IGDCC3*	< 0.001	NS	0.0362	NS	0.0280	NS	0.0298
*WNT6*	< 0.001	0.0016	0.0028	< 0.001	< 0.001	NS	NS
*ZFP42*	< 0.001	0.0011	< 0.001	0.0026	< 0.001	NS	NS

NS: not significant. Boldface gene denotes statistical significance < 0.05 for all pairwise combinations of CDDP-resistant and -sensitive TGCT cells lines.

### Down-regulated genes

*SOX2*, *NANOG* and *POU5F1* were all significantly down-regulated in all CDDP-resistant compared with all -sensitive TGCT cell lines. When CDDP-resistant cell lines were compared only with H12.1, significant down-regulation of expression was observed for the *L1TD1*, *NTF3*, *IGFBP2*, *IGFBP7*, *DNMT3L*, *ZFP42* and *WNT6* genes. In case of *GAL*, only 1777NRpmet did not show any significant change in its expression level, while both H12.1D and 1411HP showed significantly down-regulated *GAL* gene expression (*p* = 0.04 and *p* < 0.001, respectively). Similarly, 1411HP was the only CDDP-resistant TGCT cell line, which did not display change in the expression level of the *IGDCC3* gene, while in both H12.1D and 1777NRpmet significant down-regulation of its expression (*p* < 0.001 and *p* = 0.036, respectively) was demonstrated. In case of *C11orf96*, only H12.1D cell line brought its significant down-regulation (*p* = 0.02).

**Figure 5:**
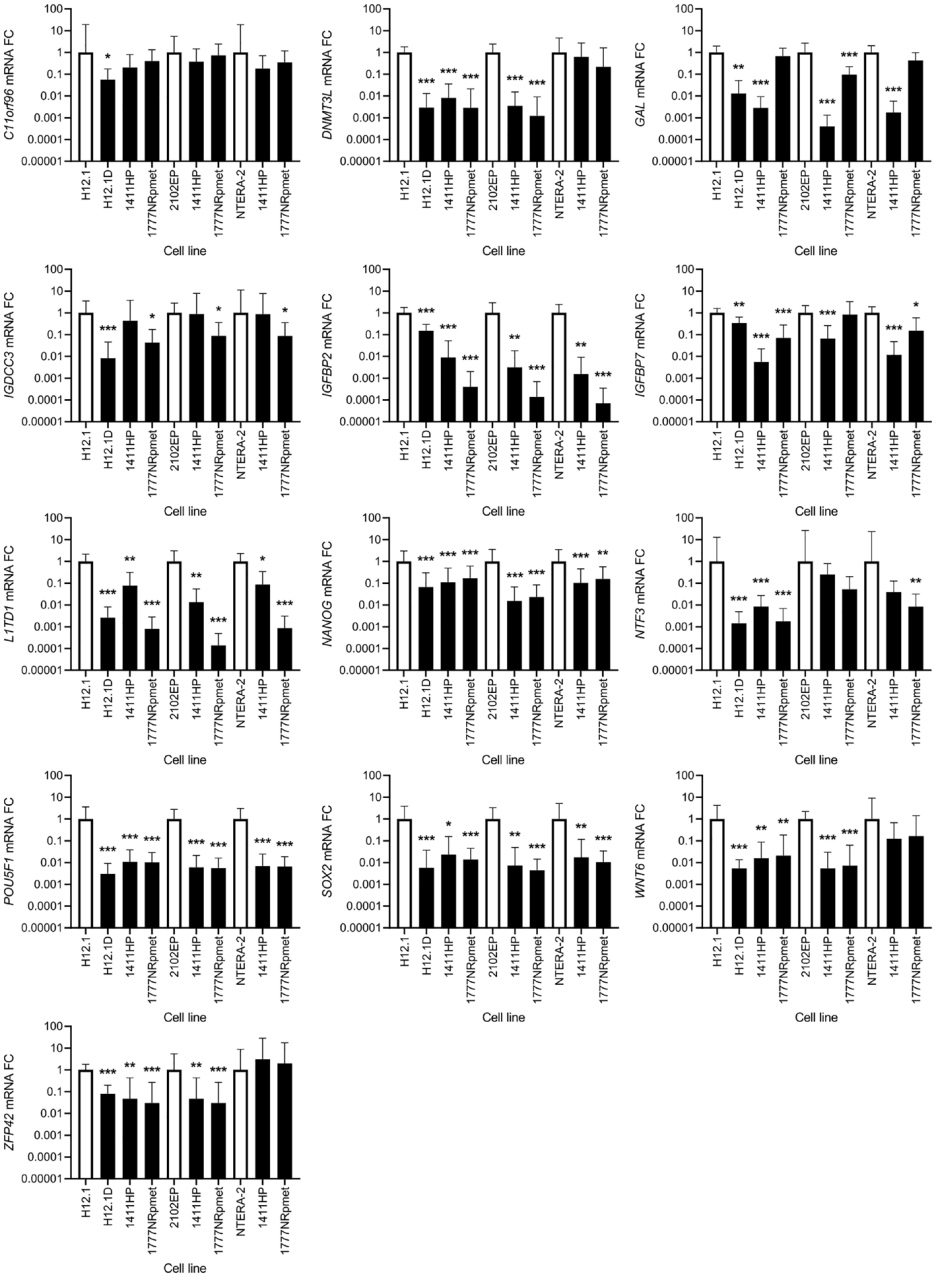
Expression change of 13 candidate genes that were originally identified as down-regulated using gene expression array. CDDP-resistant (H12.1D, 1411HP and 1777NRpmet) and -sensitive (H12.1, 2102EP and NTERA-2) TGCT cell lines are represented as black and white bars, respectively. Error bars represent upper and lower limits of expression of three technical and three biological replicates. ^*^ *p* ≤ 0.05, ^**^ *p* ≤ 0.01, ^***^ *p* ≤ 0.001.

Down-regulation of the *NTF3*, *IGFBP2* and *IGFBP7* genes was observed when their expression in two CDDP-sensitive TGCT cell lines (2102EP and NTERA-2) was compared with that in all -resistant cell lines, although data for both -resistant cell lines for *NTF3* and for 1777NRpmet for *IGFBP7*, when pairwise combined with 2102EP, were not statistically significant. *GAL* gene expression was down-regulated in both 1411HP and 1777NRpmet when compared with 2102EP (*p* < 0.001), and in 1411HP, but not in 1777NRpmet, when compared with NTERA-2 (*p* < 0.001 and *p* = 0.175, respectively). Expression of *C11orf96* was not significantly changed in 1411HP and 1777NRpmet when compared with both 2102EP and NTERA-2. *DNMT3L* expression was significantly down-regulated in both 1411HP and 1777NRpmet when compared with 2102EP (*p* < 0.001), but not if compared with NTERA-2 (*p* = 0.104). *WNT6* gene expression was down-regulated in 1411HP and 1777NRpmet TGCT cell lines in comparison with 2102EP (*p* < 0.001), but it did not significantly change in neither of them when compared with NTERA-2 (*p* = 0.112). *ZFP42* displayed identical expression change when the same cell lines were pairwise combined. In case of *IGDCC3* gene expression, significant down-regulation was observed only in case of 1777NRpmet vs 2102EP and NTERA-2 pairwise combinations (*p* = 0.028 and *p* = 0.03, respectively) (Figure 5).

## DISCUSSION

In this work, we compare gene expression profiles between the CDDP-resistant and -sensitive TGCT cell lines using gene expression array. As TGCTs are a well-curable malignity due to their extraordinary response to CDDP, our primary aim was to reveal the factors that are responsible for their sensitivity to this drug. Furthermore, we wished to disclose potential biomarkers of CDDP response that could be translated into clinical practice and could improve (either alone or in combination with IGCCCG criteria) timely and precise identification of poor prognosis TGCT patients.

We identified 6 genes that showed an opposite regulation in metastasis-derived cell lines, 1411HP and 1777NRpmet (down-regulation), compared to primary tumor-derived H12.1D (up-regulation) cell line, suggesting their regulatory role in metastatic progression of TGCT disease likely *via* tumor suppressor role. In line with this assumption, *LYPD1* has been reported to be a putative tumor suppressor gene because its overexpression in cancer cell lines reduces cell growth likely *via* induced apoptosis [48]. The *PRKCDBP* tumor suppressor gene has been shown to be: (i) significantly down-regulated in both breast cancer brain metastases and primary brain cancer with brain relapse compared to primary tumors without relapse or bone metastasis [49]; (ii) altered in colorectal cancer [50]; and (iii) contributing to the malignant progression of several tumor types [51–54].

25 genes were selected from gene expression array data set and validated by RT-qPCR. As previously reported, pluripotency factors *POU5F1*, *NANOG* and *SOX2* are significantly down-regulated in resistant TGCT cell lines and lost during disease progression towards differentiated tissues, TE, YST or CC [55–57]. Notably, down-regulation of *POU5F1*, *NANOG* and *SOX2* gene expression in CDDP-resistant TGCT cell lines was consistent, and comparable, in both gene expression array and RT-qPCR data sets, confirming previous findings. Accordingly, analysis of *POU5F1* on the protein level clearly separates sensitive TGCT cell lines showing high *POU5F1* expression in resistant TGCT cell lines lacking *POU5F1* expression [34, 57]. A recent study investigating the differentiation-dependent regulation of expression of human endogenous retrovirus K sequences (HERVK) using these TGCT cell lines confirmed *POU5F1* expression pattern and showed high *HERVK* expression in *POU5F1*-expressing sensitive EC cells (e.g., H12.1), whereas the *POU5F1*-negative, resistant cells could be further distinguished into cells with extra-embryonic differentiation characteristics towards YST (e.g., 1411HP) showing intermediate level of HERVK, and cells with somatic differentiation characteristics (1777NRpmet and H12.1D) showing low *HERVK* expression [58]. Therefore, in accordance with pluripotency markers, additional factors found in the present study to be down-regulated in all resistant TGCT cell lines may reflect the loss of the pluripotent state in general (*DNMT3L*, *WNT6* and *ZFP42*), whereas factors which also differ among resistant cell lines (*GAL*, *IGDCC3*, *IGFBP2*, *IGFBP7*, *L1TD1* and *NTF3*) may further reflect a differentiation towards different lineages. In either case, this is associated with CDDP-resistance.

*C11orf96* is an open reading frame, considered as a potential prognostic marker in colorectal cancer [59]. The *DNMT3L* gene was also classified as a prognostic marker in cervical tumors and GCTs. It is essential for germ cell development, being highly expressed in germ cells and undifferentiated pluripotent stem cells under normal developmental processes, indicating that it is also expressed in germ cell-derived tumors, and is essential for growth of human embryonal cells. Decrease of its expression could be a consequence of hypomethylation of its promoter region, and de-regulation of these processes may lead to CDDP-resistance [60, 61]. *IGDCC3* is also potential prognostic marker that is associated with overall survival (OS) and risk of recurrence in breast cancer [62]. Both IGFBP2 and IGFBP7 are members of the IGFBP family, which affect cell proliferation and migration. IGFBP2 inhibits expression of E-cadherin in colorectal cancer [63] and promotes immunosuppression associated with its mesenchymal induction in glioblastoma [64]. IGFBP7 inhibits cell proliferation and cell cycle progression [65]. Higher expression of the *IGFBP7* gene in cholangiocarcinoma was associated with better OS [66]. Low *IGFBP7* expression is a feature of leukemic stem cells associated with reduced chemotherapy sensitivity [67], suggesting that its expression levels can be considered as a prognostic marker of chemoresistance. *L1TD1* is highly expressed gene in pluripotent cells and is important for maintaining the pluripotent state. It has been shown to be controlled by *POU5F1*, *NANOG* and *SOX2* [68, 69], an observation indirectly paralleled by our data. Hence, it seems that L1TD1 might represent marker for phenotyping and monitoring of TGCT progression. *WNT6* promotes chemoresistance in various cancer cells, as it is up-regulated by chemotherapeutics, thereby enhancing tumor resistance, aggressiveness and progression [70, 71, 72]. Along with SOX2, POU5F1, NANOG, SSEA-3 and SSEA-4, ZFP42 is an embryonic marker [73]. *NOTCH3* amplification leads to increased expression of genes associated with embryonic stem cell development (*NANOG*, *POU5F1* and *ZFP42*), ultimately leading to chemoresistance development [74]. Our results suggest that decreased expression of *ZFP42* in CDDP-resistant TGCT cell lines in comparison to two CDDP-sensitive cell lines might parallel an *L1TD1* situation, considering that *SOX2*, *NANOG* and *POU5F1* expression was also significantly down-regulated, indicating that effect of NOTCH3 on these embryonic markers is deregulated in CDDP-resistant TGCT cell lines.

We found no significant difference in the level of BEX2, a protein that promotes proliferation in colorectal cancer [75] and regulates cell proliferation and migration, invasion and apoptosis in malignant glioma [76, 77]. Obviously, it does not have a similar effect in GCTs, as demonstrated herein. We found increased expression of the *CYB5A* and *CYB5R2* genes in all CDDP-resistant TGCT cell lines when compared to H12.1 -sensitive cell line. CYB5A has a key role in lipid-radical cycles in membranes, leading to positive effects on microsomal and mitochondrial oxidation [78]. CYB5R2 is a cytochrome b5 reductase 2, methylation of which in nasopharyngeal carcinoma was associated with lymph node metastasis [79]. The increased expression observed in the present study might suggest a role of CYB5A and CYB5R2 in metabolic alterations causing CDDP-resistant phenotype and metastatic progression in TGCTs. Since *CCN1* showed a similar trend in the expression level in metastasis-derived cell lines, it may also play a role in metastatic spread in TGCTs. Accordingly, it has been reported to act as a key inducing factor in the metastatic progression and chemoresistance in some other cancer types [80, 81]. FADS2 is a protein involved in an unknown plasticity of the lipid metabolism of some tumor types [82], suggesting another metabolism modification leading to chemo-resistant phenotype in TGCTs. *ID2* was significantly overexpressed in all CDDP-resistant TGCT cell lines. In other cancers, the ID2 protein promotes early-stage progression [83] and survival during metabolic stress [84], and its defect leads to a more differentiated less aggressive phenotype [85]. We found no significant changes in *ISG20* expression, although ISG20 is a protein whose forced expression leads to significant promotion of metastasis and angiogenesis in hepatocellular carcinoma [86]. The consistent overexpression of *PCP4* in all CDDP-resistant TGCT cell lines may parallel observation showing that PCP4 regulates apoptosis in breast cancer cells [87]. We revealed no significant difference in expression of *REC8*, which has tumor suppressive effects partially mediated by down-regulation of genes involved in cell growth and up-regulation of apoptosis/migration inhibitors [88], accounting for the lack of its tumor suppressive effect in TGCTs. *SLC40A1* expression was consistently up-regulated in all resistant TGCT cell lines. SLC40A1 is an iron exporter possessing many putative Nrf2 binding sites. Elevated levels of Nrf2 and reduced levels of SLC40A1 were previously found in CDDP-resistant ovarian cancer cells [89], contradictory to our findings, indicating different iron needs and transport modification in TGCTs. Hence, the increased expression of iron transporters may contribute to CDDP-resistance *via* pre-target mechanism of resistance [14] in this malignity, where copper transporters ATP7A and ATP7B are overexpressed and contribute to CDDP-resistance [90]. However, further work is required to verify this assumption. We found significantly increased expression of *TMSB4X* in 1411HP and 1777NRpmet, but decreased expression in H12.1D TGCT cell line, compared to H12.1. In ovarian cancer, increased levels of this protein play a role in accelerating proliferation, invasion and migration [91]. This protein is also a candidate biomarker in head and neck squamous cell carcinomas [92]. According to our results, TMSB4X might be able of discriminating between the intrinsic and acquired resistance, as it was up-regulated only in metastasis-derived TGCT cell lines, which were isolated from patients with multidrug/intrinsic resistance towards CDDP. TRIB3 plays an important role in cancer by inhibiting proliferation, as *TRIB3* silencing significantly inhibited HaCaT cell proliferation [93], knockdown inhibited lung cancer cell migration, invasion, epithelial-mesenchymal transition and stemness [94], and its elevated levels supported breast cancer progression [95]. As it was up-regulated in all CDDP-resistant TGCT cell lines, its role in TGCT cell proliferation and migration is also highly expected.

Of course, we are aware of the fact that our study has some limitations. These result mainly from the combination of cell lines used, as only one pair of TGCT cell lines originates from the same cell clone and resistant derivative was not really made CDDP-resistant, for instance by cultivation with sub-lethal doses of this drug, as used in recent works in the field [96–98]. Instead, the parental CDDP-sensitive cell line was *in vitro* differentiated using differentiation-inducing medium. Other pairs combine non-isogenic cell lines. In general, differentiation or losing the embryonal pluripotent state does induce CDDP resistance. However, in the case of H12.1D, the differentiation is towards somatic lineages, and therefore more resembles teratoma, which is not that clinical problem. The 1411HP cells have lost the pluripotent state, are CDDP resistant and show malignant growth in nude mice. The tumor consists of undifferentiated carcinoma with EC-like morphology and differentiation features of YST. We propose that it is an extra-embryonal progenitor [46]. It more represents the clinically relevant CDDP resistant malignant TGCT type. Both metastasis-derived TGCT cell lines represent models with natural (true) resistance which can also be considered as an advantage compared to the models with artificially established resistance. With the 1411HP, we investigated a model which can represent a “resistant non-teratoma tumor”. Nevertheless, resistance marker found in H12.1D might be useful to investigate resistance mechanisms in other cells.

## MATERIALS AND METHODS

### Cell cultures

H12.1, 2102EP, H12.1D, 1411HP and 1777NRpmet TGCT cell lines (Table 1) were grown in RPMI-1640 medium supplemented with 10% fetal bovine serum (FBS), penicillin (100 units/ml) and streptomycin (10 μl/ml). NTERA-2 TGCT cell line (Table 1) was grown in Dulbecco’s modified eagle’s medium supplemented with F-10 nutrient mixture (1:1), 10% FBS, penicillin (100 units/ml) and streptomycin (10 μl/ml). Cell lines were cultivated at 37ºC in 5% CO_2_ atmosphere.

### MTT assay

Viability of TGCT cell lines after CDDP treatment was determined by the MTT assay. Briefly, 5 × 10^3^ cells were seeded into 96-well plate and allowed to grow/attach for 24 hr. The cells were treated with the increasing concentrations of CDDP in culture medium for 24 hr. Cells were then washed with PBS and MTT (1 mg/ml) was added to cell suspensions. The cells were incubated with MTT for 4 hr. Afterwards, MTT was discarded, and DMSO was added to dissolve formazan crystals. The resulting absorbance was measured at wavelength of 540 and 690 nm with xMark^™^ Microplate Spectrophotometer (Bio-Rad Laboratories, Inc.). The IC_50_ values were determined by GraphPad Prism (version 8.4.3).

### RNA extraction

To extract the total RNA, TRI Reagent solution (Life Technologies) was used. Total RNA was quantified using MaestroNano Spectrophotometer (Applied Biological Materials Inc.) and Qubit fluorometer (Qubit^®^ RNA HS Assay Kit, Life Technologies). RNA integrity was assessed using the Agilent 2100 bioanalyzer (Agilent, Palo Alto, CA, USA). Extracted RNA was used for microarray analysis and RT-qPCR validation.

### Gene expression profiling

Genome-wide gene expression analysis was performed using Agilent SurePrint G3 Human Gene Expression v3 8x60K Microarray kit (product # G4851C, design ID 072363, Agilent Technologies). Each array contained 60-mer oligonucleotide probes for 26,083 unique genes/transcripts and 30,606 unique lncRNAs. One-color microarray-based gene expression analysis was conducted according to the manufacturer’s instructions. Briefly, 200 ng of total RNA from each sample was used. After annealing of the T7 promoter primer to the total RNA, the samples were incubated and labelled with Cy3-CTP to perform reverse transcription according to the original protocol (One-Color Microarray-Based Gene Expression Analysis ver.6.9, G414090040). The labelled samples were hybridized onto the SurePrint G3 Human Gene Expression microarray in the Agilent Microarray Hybridization Chamber (G2534) for 20 h at 65°C/20 rpm in a rotator oven. After washing with the Gene Expression Wash Buffers 1 and 2 (5188–5327) and drying, slides were scanned in the Innoscan 900 microarray scanner (Innopsys Inc., Carbonne, France) with the resolution of 3 micrometers. The resulting images of individual arrays were extracted with the Mapix 7.3.0 software (Innopsys Inc., Carbonne, France) and exported as GPR files. For each TGCT cell line, gene expression arrays were repeated for 3 individual biological replicates.

To determine gene expression from GPR files, correction of background/signal to noise ratio was performed to output values, and probe signal summarization to the level of individual gene expression was applied. In the next step, the corrected differentially expressed genes were identified by limma package, and FC of gene expression in CDDP-resistant vs -sensitive TGCT cell lines were calculated, along with average expression and *p* values, in order to identify difference in gene expression of CDDP-resistant TGCT cell lines.

For result interpretation, we consider gene expression to be up-regulated, when ratio in the mean mRNA expression/FC of CDDP-resistant vs -sensitive TGCT cell line was > 1, and down-regulated, when the mean FC between CDDP-resistant and -sensitive TGCT cell line was < 1. This is due to the fact, that after microarray visualization, and correction to background noise, we compared the corrected resulting signals (representing gene expression in samples) as proportion/difference in average expression in CDDP-resistant TGCT cell line vs -sensitive one (H12.1).

### RT-qPCR validation

The altered expression of the selected genes identified in microarray analysis was validated using SYBR Green-based qPCR. Differentially expressed genes were evaluated by RT-qPCR using First-Strand cDNA Synthesis System (Central European Biosystems, Czech Republic). Briefly, for cDNA synthesis in a reaction of a final volume of 20 μl, 1,500 ng of total RNA, 2 μl of 10x MuLV buffer, 1 μM of p(dN)6 primer, 0.1 mM of dNTP mixture and 100 units of MuLV reverse transcriptase were incubated at 42°C for 1 hr followed by enzyme inactivation at 95°C for 5 min. RT-qPCR detection and quantification of the selected genes and the *PGK1* and β-actin reference genes was performed using SYBR Premix Ex Taq II (Tli RNaseH Plus), ROX plus (Takara, Japan) and specific reverse and forward primers listed in Supplementary Table 9. qPCR was performed using Agilent, ARIA Real-Time PCR System (Agilent) at following setting: 95°C for 5 min, followed by 40 cycles of 95°C for 20 sec and 60°C for 50 sec, followed by melt cycle. All samples were amplified in triplicates. Ct values were averaged and normalized against reference endogenous genes *PGK1* and β-actin, stably expressed across all TGCT cell lines tested. The average Ct value of *PGK1* was 22.8 ± 1.29 for CDDP-sensitive and 23.05 ± 1.58 for -resistant TGCT cell lines; average Ct value for β-actin in CDDP-sensitive TGCT cell lines was 17.18 ± 0.98 and for -resistant TGCT cell lines 16.67 ± 0.81.

### Statistical analysis

For statistical analysis of the gene expression data, SigmaPlot 12.5 and Prism GraphPad 8.4.3 were used. Normality of distribution was tested by the Shapiro-Wilk test. Relative quantification of the gene expression was calculated with 2^-ΔΔCt^ method, which represents relative FCs of expression. Therefore, ΔΔCt = ΔCt (CDDP-resistant cell line) - ΔCt (CDDP-sensitive cell line). Analysis of the significance of FC in the gene expression between the studied groups was applied to the ΔCt values. If normally distributed, gene expression data were tested by analysis of variance (ANOVA) with the Bonferroni’s post-hoc test for multiple comparisons. If data were normally distributed but did not pass Levene’ test for equality of variances, One-way ANOVA was used with Tamhane’s T2 test for multiple comparisons. If the data were non-normally distributed, Kruskal-Wallis One Way ANOVA with Dunn’s test for multiple comparisons was used. All tests were two-tailed, performed at the significance level α = 0.05. For all analyses, *p* < 0.05 was considered statistically significant (^*^ *p* < 0.05; ^**^ *p* < 0.01, ^***^ *p* < 0.001). Graphic output in the form of a heatmap for gene expression microarray analysis was performed using RStudio (RStudio, Inc.; version 1.2.1335). The cut-off of significantly up- or down-regulated genes was selected according to their log FC and adjusted *p* value in above-mentioned software and imported into RStudio. Heatmap dendrogram creation parameters were set based on data similarity.

To conduct predictive interactome analysis, STRING v 11 [99] was used (https://string-db.org). Out of 281 genes (the overlapping genes from gene expression microarray, which had > 2 FC and adjusted *p* value < 0.05), 247 proteins were recognized by the STRING database. Interactome settings were as follows: (i) meaning of network edges was set to molecular action (line shape indicates the predicted mode of action); (ii) interaction channels, i.e., text-mining, experiments, databases, co-expression, neighborhood, gene fusion and co-occurrence, were active; and (iii) minimum required interaction score was set to high confidence (0.700).

## CONCLUSIONS

**Figure 6:**
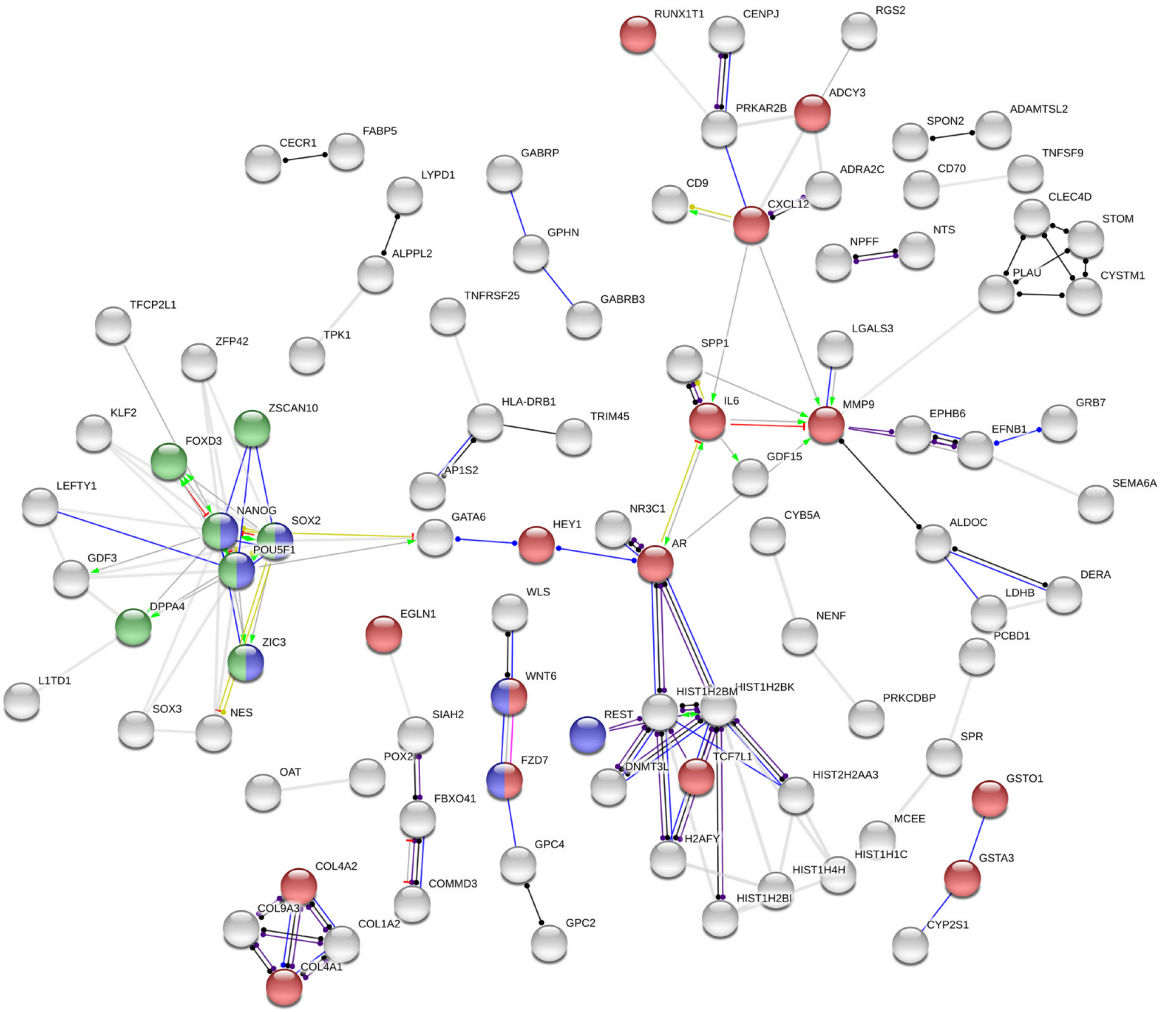
Association network in STRING. Out of 281 genes, 247 proteins were recognized by the STRING database and additional 152 were hidden as disconnected nodes, when the confidence level has been set to ‘high’ (0.700). Coloured nodes represent at least one of the three selected functional enrichment network categories: (i) pathways in cancer (KEGG pathways database; red); (ii) signaling pathways regulating pluripotency of stem cells (KEGG pathways database; blue), and (iii) transcriptional regulation of pluripotent stem cells (Reactome pathways database; green). Action types: activation (green), binding (blue), inhibition (red), phenotype (cyan), reaction (black), catalysis (purple), post-translational modification (magenta) and transcriptional regulation (yellow). Action effects: positive (arrow; →), negative (capped line; ┴) and unspecified (dot; ●).

In the present study, we compared gene expression profiles in the group of CDDP-resistant and -sensitive TGCT cell lines using gene expression array. We identified 281 genes, which were significantly de-regulated in CDDP-resistant compared to -sensitive TGCT cell lines. Of them, 247 were recognized by the STRING database. Based on the generated association, three main categories were identified as important for CDDP response in TGCT cells, pathways relevant for cancer in general, signaling pathways regulating pluripotency of stem cells, and transcriptional regulation of pluripotent stem cells (Figure 6). 25 genes with the highest expression change were validated using RT-qPCR. The *DNMT3L*, *GAL*, *IGFBP2*, *IGFBP7*, *L1TD1*, *NANOG*, *NTF3*, *POU5F1*, *SOX2*, *WNT6*, *ZFP42*, *ID2*, *PCP4*, *SLC40A1* and *TRIB3* genes displayed comparable expression change in both gene expression array and RT-qPCR experiment. We propose that the identified genes, whose expression is stably changed across all CDDP-resistant TGCT cell lines, independently of the origin of resistance, could after clinical validation serve as prognostic markers for early detection of CDDP response and for timely and precisely treatment optimization.

## SUPPLEMENTARY MATERIALS





## Abbreviations

TGCT: Testicular germ cell tumor
CDDP: cisplatin
SE: seminoma
NSE: non-seminoma
EC: embryonal carcinoma
CC: choriocarcinoma
YST: yolk sac tumor
TE: teratoma
DDR: DNA damage response
NER: nucleotide excision repair
AFP: α-fetoprotein
IGCCCG: International Germ Cell Cancer Cooperative Group
MTT: (3-(4,5-dimethylthiazol-2-yl)-2,5-diphenyltetrazolium bromide
FBS: fetal bovine serum
FC: fold change
ANOVA: analysis of variance
IC: inhibitory concentration
GCT: germ cell tumor
HERVK: human endogenous retrovirus K sequences
DMSO: dimethyl sulfoxide
