# Supplementary material for: Differential gene expression in cisplatin-resistant and -sensitive testicular germ cell tumor cell lines
Source: Oncotarget. 2020 Dec 22;11(51):4735–53. doi: 10.18632/oncotarget.27844 (PMC7771712; doi:10.18632/oncotarget.27844)
Supplement: Supplementary file 1 [file oncotarget-11-4735-s001.pdf]

Differential gene expression in cisplatin-resistant and -sensitive testicular germ cell tumor cell lines

SUPPLEMENTARY MATERIALS

Supplementary Table 1: List of genes displaying statistically significant change in expression between the H12.1D and H12.1 TGCT cell lines. See Supplementary Table 1

Supplementary Table 2: List of genes displaying statistically significant change in expression between the 1411HP and H12.1 TGCT cell lines. See Supplementary Table 2

Supplementary Table 3: List of genes displaying statistically significant change in expression between the 1777NRpmet and H12.1 TGCT cell lines. See Supplementary Table 3

Supplementary Table 4: List of genes displaying statistically significant change in expression between the H12.1D and H12.1 TGCT cell lines (FC > 2.0 and < 0.5 for up- and down-regulated expression, respectively). See Supplementary Table 4

Supplementary Table 5: List of genes displaying statistically significant change in expression between the 1411HP and H12.1 TGCT cell lines (FC > 2.0 and < 0.5 for up- and down-regulated expression, respectively). See Supplementary Table 5

Supplementary Table 6: List of genes displaying statistically significant change in expression between the 1777NRpmet and H12.1 TGCT cell lines (FC > 2.0 and < 0.5 for up- and down-regulated expression, respectively). See Supplementary Table 6

Supplementary Table 7: Overlap of data sets shown in Supplementary Tables 4, 5 and 6. See Supplementary Table 7

Supplementary Table 8: List of the 25 most differentially expressed genes between the CDDP-resistant and -sensitive TGCT cell lines. See Supplementary Table 8

Supplementary Table 9: Primers for validation of the 25 most differentially expressed genes between the CDDP-resistant and -sensitive TGCT cell lines

Gene	Forward (5′–3′)	Reverse (5′–3′)
BEX2	CGAAAGTAGGAAGCGGAGG	GACTCCATTACTCCTGGGC
CYB5A	ACAGACCAAAGTTAAACAAGCCTC	AGGCGATACATCAAGGCGAC
CYB5R2	AATTGCCAGGACTCACCCAG	GACTTCGCTGGAGGAGGAAG
CYR61/CCN1	CCGCCTTGTGAAAGAAACCC	CTTGCCCTTTTTCAGGCTGC
PCP4	TGGGGCAACCAATGGAAAAG	GTCTCTGGTGCATCCATGTC
ISG20	CTTGAATCCTGTGGGTCCAAAATG	GATGCAACAGCAAAGGGTGG
REC	CGAAAGTAGGAAGCGGAGG	GACTCCATTACTCCTGGGC
TRIB3	GACCGTGAGAGGAAGAAGC	CCACAGGGAATCATCTGGC
FADS2	CACTACGCTGGAGAAGATGC	TCTTTGAGTTCTTGCCGTGG
ID2	GCAGGCTTCTGAATTCCCTTC	TGAACACCGCTTATTCAGCC
TMSB4X	AGACTTCGCTCGTACTCGTG	TCGATCTCAGCCATATCGGG
SLC40A1	CTGTTTGCAGGCGTCATTG	ATTTTCTTGCAGCAACTGTGTC
IGFBP2	CCGGAGCAGGTTGCAG	CGGCCAGCTCCTTCATAC
IGFBP7	TGGGTGCTGGTATCTCCTC	CTGGAGGTTTATAGCTCGGC
L1TD1	CCTCACTTTGTTCGCTCCTC	ACGTCAATCAAGTCTCAGCAG
NTF3	GGAGACTTTGAATGACCGAGC	GGACATCACCTTGTTCACCTG
NANOG	CCTCCAGCAGATGCAAGAAC	AGGAAGAGTAAAGGCTGGGG
PFOU5F1	GGAGAAGCTGGAGCAAAACC	TTTGGCTGAATACCTTCCCAAATAG
SOX2	CTACAGCATGATGCAGGACC	CGTTCATGTAGGTCTGCGAG
C11orf96	GCATCTGCTCCAGTTACCAG	TGGATCTCGTCGAAGGTCAC
DNMT3L	GCATGGACGTGATTTTGGTG	GTGTGAACCTGGAGACTTCC
GAL	AACCAGGAAGCTTTGACAGG	GATGTCTTCTGAGGAGGCTG
IGDCC3	GGCACAGGAAACCTCATCATC	GGGATGCTGCACAAACTCAG
WNT6	GTGCAACTGCACAACAACG	CGAAATGGAGGCAGCTTCTG
ZFP42	ACCTAACCATCGCTGAGCTG	TATCAACCACCTCCAGGCAG
